# Plasticity Induced by Intermittent Theta Burst Stimulation in Bilateral Motor Cortices Is Not Altered in Older Adults

**DOI:** 10.1155/2015/323409

**Published:** 2015-05-06

**Authors:** Daina S. E. Dickins, Martin V. Sale, Marc R. Kamke

**Affiliations:** The Queensland Brain Institute, The University of Queensland, St Lucia, QLD 4072, Australia

## Abstract

Numerous studies have reported that plasticity induced in the motor cortex by transcranial magnetic stimulation (TMS) is attenuated in older adults. Those investigations, however, have focused solely on the stimulated hemisphere. Compared to young adults, older adults exhibit more widespread activity across bilateral motor cortices during the performance of unilateral motor tasks, suggesting that the manifestation of plasticity might also be altered. To address this question, twenty young (<35 years old) and older adults (>65 years) underwent intermittent theta burst stimulation (iTBS) whilst attending to the hand targeted by the plasticity-inducing procedure. The amplitude of motor evoked potentials (MEPs) elicited by single pulse TMS was used to quantify cortical excitability before and after iTBS. Individual responses to iTBS were highly variable, with half the participants showing an unexpected decrease in cortical excitability. Contrary to predictions, however, there were no age-related differences in the magnitude or manifestation of plasticity across bilateral motor cortices. The findings suggest that advancing age does not influence the capacity for, or manifestation of, plasticity induced by iTBS.

## 1. Introduction

Plasticity is the ability of the brain to undergo enduring morphological and functional change in response to the demands of its environment [1]. In humans, transcranial magnetic stimulation (TMS) can be used to induce short term changes in cortical excitability that are believed to be underpinned by NMDA-dependent synaptic plasticity [2]. Studies using TMS have reported plasticity to be reduced in the primary motor cortices (M1) of older adults [3–5], but measures of excitability were limited to the hemisphere targeted by the stimulation. The current study aimed to investigate whether plasticity manifests more diffusely in older compared to younger adults.

In young adults, TMS-induced plasticity has been demonstrated to manifest not only in the stimulated cortex, but also in the unstimulated hemisphere [6–12]. For example, Shin and Sohn [12] used paired associative stimulation (PAS), which involves repeatedly pairing single pulse TMS to the cortical representation of the thumb with peripheral stimulation to the median nerve, to induce plasticity. The effects were probed using motor evoked potentials (MEPs), which are elicited by single pulse TMS and provide an indirect measure of cortical excitability, with changes in amplitude believed to reflect synaptic plasticity (see [13] for review). Following PAS, which was applied only to the left cortex, MEP amplitudes were enhanced both in the muscle targeted by the procedure and in the homologous muscle of the other hand. PAS has also been used to investigate plasticity in older adults, with results showing a reduction in plasticity [3, 4]. Similar effects have been found with plasticity induced by repetitive TMS [5], and both of these observations are consistent with demonstrations of reduced plasticity following motor training (see [14] for review) as well as with evidence of attenuated plasticity in aged nonhuman animals ([15], see [16] for review). Importantly, older adults exhibit greater and more diffuse neural activity, both between and within hemispheres, when performing similar tasks to their younger counterparts, an effect attributed to age-related neurobiological change [17–25]. Therefore it is possible that the manifestation of plasticity across the hemispheres is altered in older adults.

To date, only one study has investigated age-related differences in the manifestation of plasticity induced by TMS across the stimulated and unstimulated hemisphere. Di Lazzaro and colleagues [26] administered intermittent theta burst stimulation (iTBS), which delivers high-frequency bursts of TMS at the natural theta rhythm of the hippocampus to induce long-term potentiation- (LTP-) like plasticity [27]. They found preliminary evidence indicating that plasticity induced by iTBS in the nondominant hemisphere also manifests in the contralateral motor cortices of both young and older adults. Given evidence of age-related differences in the spread of neural activity, this result suggests that any assessment of plasticity in older adults should consider changes in both motor cortices. Critically, only six older adults were tested in that study and there was no control for the potential modulation of iTBS-induced plasticity by attention. The failure to control attention is an important limitation as older adults have been shown to experience decline in a range of cognitive abilities [28], including attention [29], and we have shown previously that attention alters plasticity induced by PAS and iTBS [30, 31].

The current study aimed to investigate the extent to which plasticity manifests across bilateral motor cortices in young and older adults following iTBS to the dominant (left) hemisphere. It was predicted that plasticity would be reduced in the stimulated hemisphere of older, relative to young, adults. It was further predicted that this reduction in older adults would be accompanied by greater plasticity in the unstimulated hemisphere, relative to young adults.

## 2. Methods

### 2.1. Participants

Twenty younger participants aged 18–28 years (*M* = 22.95,  SD = 2.52, males = 10) and 20 older participants aged 65–76 (*M* = 70.15, SD = 3.07, males = 10) completed the study. Two additional participants in the older group failed to complete sessions due to discomfort during the iTBS protocol. According to the Edinburgh handedness inventory [32], all participants were right-handed except one young and one older adult who were ambidextrous (young *M* = 77.47, SD = 19.47, and range = 33.33–100; older *M* = 84.31, SD = 17.17, and range = 33.33–100). Volunteers were recruited through advertising in online newsletters and by word of mouth and were reimbursed $10 per hour for their participation. A TMS safety-screening questionnaire [33, 34] was used to exclude volunteers with known neurological disease or damage, epilepsy, history of head injury, or psychiatric disorder, or those taking neuroactive medications. All participants had normal or corrected-to-normal visual acuity. Participants provided informed consent and all procedures were approved by The University of Queensland Medical Research Ethics Committee in accordance with the Declaration of Helsinki.

### 2.2. Transcranial Magnetic Stimulation (TMS) and Electromyography (EMG)

Changes in cortical excitability induced by iTBS were quantified using single pulse TMS. A figure-of-eight shaped coil with a wing diameter of 70 mm, connected to a Magstim 200^2^ stimulator, was used to administer monophasic TMS. The coil was angled 45 degrees from the midline with the handle pointing towards the back of the head. The coil was placed tangentially on the scalp and moved systematically in a grid-like pattern until the motor hotspot was located. The motor hotspot was defined as the optimal position on the scalp for evoking the largest and most consistent MEP (peak-to-peak amplitude) in the target muscle, the* abductor pollicis brevis* (APB) of the left and right hands. Stimulation was applied at an intensity sufficient to evoke a clear motor response in the targeted muscle and occurred approximately every five seconds. The position of the coil for each hotspot was recorded using a frameless infrared stereotaxic neuronavigation system (Visor 1, ANT, Netherlands), which was used to reproduce coil angle and location within an experimental session.

Following determination of the hotspot, resting motor thresholds (rMTs) were obtained for the cortical representation controlling the left and right APB. rMT was defined as the minimum TMS intensity that evoked an MEP of above 50 *μ*V in at least three out of five consecutive trials. The intensity of the TMS was adjusted using a staircase (two-down, one-up) procedure until the criterion was met. Test TMS intensities for the left and right APBs were then established, defined as that required to evoke an average MEP of approximately 1 mV (peak-to-peak) in the resting muscles. To determine average MEP amplitude at baseline and after iTBS, TMS pulses were delivered at the test intensity every 5 ± 1 seconds for a total of 21 pulses.

Surface electromyography (EMG) was employed to record activity from the APB muscles using disposable 24 mm silver-silver chloride (Ag/AgCl) electrodes in a belly-tendon montage. MEP data were amplified (×1000), filtered (20–1000 Hz), and sampled at 2000 Hz using a NeuroLog System (Digitimer, UK). Data were stored for offline analysis using Signal software (CED, UK).

### 2.3. Intermittent Theta Burst Stimulation (iTBS)

Plasticity was induced using the standard iTBS protocol, administered with a Magstim Super Rapid^2^ stimulator. A burst of three high-frequency TMS pulses (50 Hz) was repeated at a frequency of 5 Hz for two seconds (10 bursts in total). This train was followed by an eight-second rest period [27]. Stimulation continued in this format until a total of 600 pulses were administered. iTBS was delivered to the left hemisphere at 70% of rMT. This intensity was based on the rMT measured with the Magstim Super Rapid^2^ stimulator immediately prior to the iTBS intervention.

### 2.4. Attention Task

**Figure 1:**
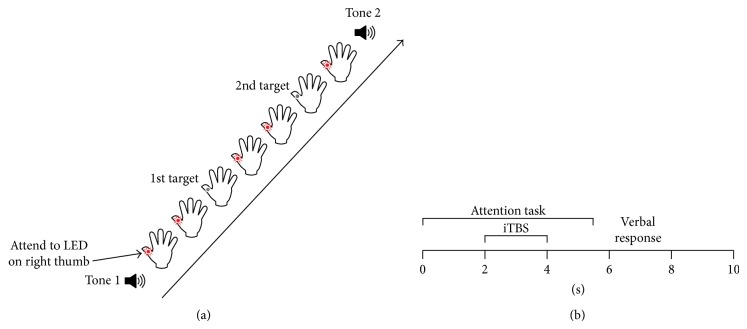
Attention task and trial timeline. (a) Attention task. Participants were instructed to attend to a continuously lit LED attached to the right thumb and to count the number of interruptions (“OFF” periods). A tone indicated the start and end of each trial. (b) Trial timeline. Each trial lasted a total of 10 seconds, with iTBS occurring during the attention task.

In order to control for any modulatory effects of attention on plasticity [30, 31], participants overtly attended to a light-emitting diode (LED) attached just above the interphalangeal joint of the right thumb during the iTBS intervention. Participants were tasked with detecting the number of brief interruptions (“OFF” periods) to the continuously lit LED. Specifically, as presented in Figure 1, each trial began with the presentation of a high pitch tone, after which participants attended to the LED and silently counted the number of “OFF” periods. The presentation of a low pitch tone after iTBS signalled the end of the trial, which lasted 5.5 seconds, and prompted participants to make a verbal response as to the number of “OFF” periods detected. There could be 0, 1, or 2 “OFF” periods in a trial and these occurred between 2 and 5.5 seconds (see Figure 1). The number of “OFF” periods presented in a single trial was randomised and the time at which each “OFF” period occurred was jittered. When two targets were present in a trial, the second target always appeared after the iTBS burst, which occurred between 2 and 4 seconds. The task consisted of 20 trials in total.

### 2.5. Experimental Design and Procedure

**Figure 2:**
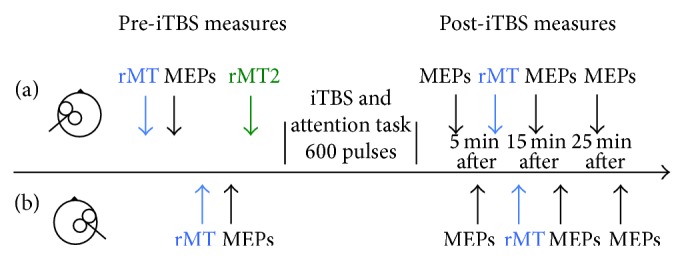
Experimental timeline. The timeline of measures is shown for the left (stimulated) hemisphere in the upper panel (a) and the right (unstimulated) hemisphere in the lower panel (b). Cortical excitability was probed before and after intermittent theta burst stimulation (iTBS) using resting motor threshold (rMT) and motor evoked potential (MEP) amplitude. Transcranial magnetic stimulation (TMS) targeted the representation in motor cortex of the right and left abductor pollicis brevis muscle. iTBS targeted the left (i.e., “stimulated”) hemisphere only. The rMT2 measure was used to establish stimulation intensity for iTBS (see Section 2.3).

Participants were seated comfortably with both arms and hands resting on cushioned platforms on a desk. As shown in the time course for the experiment in Figure 2, single pulse TMS was applied to the left and right M1 to locate the motor hotspot and to quantify cortical excitability before the iTBS intervention. Following this, the participant was provided with a brief practice of the attention task and the iTBS intervention was then administered concurrently with the spatial attention task. Cortical excitability was measured with single pulse TMS 5, 15, and 25 minutes following iTBS. At each time point, MEPs were first obtained from the left (stimulated) M1 followed by the right (unstimulated) M1 using the test TMS intensity (see Figure 2). Participants were monitored throughout the experiment to ensure their eyes remained open and their hands and arms remained relaxed at all times.

### 2.6. Data Processing and Analyses

Performance on the spatial attention task was quantified by the number of correct trials and was compared between young and older adults using an independent samples *t*-test. For the plasticity effects, the first trial from each block of MEP data (21 MEPs per block) was removed. Trials with voluntary muscle activity evident in the 100 ms prior to TMS (totaling 2.3% of remaining trials) were also removed and the remaining trials were averaged. Baseline rMTs, MEPs, and test stimulus intensities were separately subjected to a 2 × 2 mixed ANOVA with the repeated measures factor of hemisphere (stimulated, unstimulated) and the between-subjects factor age (young, older). Post-iTBS MEP amplitudes were normalized to baseline and assessed using a mixed ANOVA with the repeated measures factors of time (5, 15, and 25 minutes after) and hemisphere (stimulated, unstimulated) and the between-subjects factor of age (young, older).

Exploratory analyses were conducted separating participants into two groups: participants demonstrating increases in average MEP amplitude (average MEP change > 0%) in the right APB after iTBS (LTP-like responders) and participants demonstrating decreases in average MEP amplitude (average MEP change < 0%) in the APB after iTBS, similar characteristically to long-term depression (LTD-like responders) [27]. The factor response type (LTP-like, LTD-like) was then added to the original ANOVA. Bonferroni corrections were applied to all follow-up, two-tailed tests.

## 3. Results

### 3.1. Performance on the Spatial Attention Task

Both groups performed very highly on the attention task, with an average of 19.40 (±0.94; *M* ± SD) out of 20 correct responses for the young and 19.80 (±0.41) for the older adults. Accuracy did not differ significantly between the two age groups (*t*(38) = 1.74, *p* = 0.093). This result suggests that any age differences in plasticity following iTBS are unlikely to be due to systematic variation in the allocation of spatial attention.

### 3.2. Baseline Cortical Excitability

Resting motor thresholds, baseline MEPs, and test stimulus intensities for the stimulated and unstimulated hemispheres in young and older adults are shown in Table 1. There was little difference between groups in rMTs at baseline, with ANOVA failing to reveal any significant effects (all *p*'s > 0.125). Average raw MEP amplitudes also did not differ significantly at baseline (all main effects and interactions: *p* > 0.147). Finally, although average test stimulus intensity was slightly lower in the young compared to older adults, which was confirmed by a main effect of age (*F*(1,38) = 6.05, *p* = 0.019, and *ηp* ^2^ = 0.137), test stimulus intensities did not differ significantly between the hemispheres, as indicated by the absence of any additional main effects or interactions (all *p*'s > 0.732).

### 3.3. iTBS-Induced Plasticity in Bilateral Motor Cortices of Young and Older Adults

**Figure 3:**
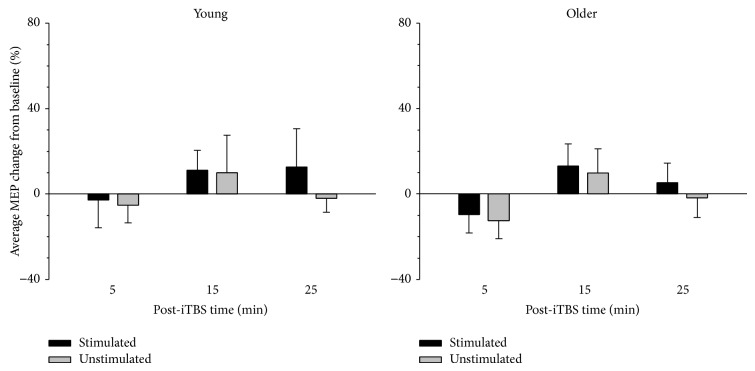
Average normalised MEP change following iTBS. Average MEP change relative to each individual's baseline varied significantly across post-iTBS measures with the largest difference evident between the 5- and 15-minute time points. This pattern was comparable between young and older adults and between the hemisphere receiving iTBS (stimulated) and the hemisphere not receiving iTBS (unstimulated). Error bars denote* SEM*.

The overall change in MEP amplitude at each of the three time points following iTBS (relative to each participant's baseline) is depicted in Figure 3. MEPs decreased immediately following iTBS in both groups but increased at the 15-minute time point. This change in post-iTBS measures across the time points was reliable, as confirmed by a significant main effect of time; *F*(2,76) = 5.80, *p* = 0.009, *ηp* ^2^ = 0.132. The effect of time, however, did not vary significantly as a function of age or hemisphere (*p*'s > 0.505). Follow-up comparisons revealed a significant difference between MEP change at 5 and 15 mins after iTBS (*t*(39) = 2.78, *p* = 0.008), a marginally significant difference between 5 and 25 mins (*t*(39) = 2.48, *p* = 0.018), and no significant difference between 15 and 25 mins after (*t*(39) = 1.52, *p* = 0.136; adjusted alpha = 0.017). Although the preceding analysis revealed a difference in MEP change across the post-iTBS time points, it did not test whether any of the changes were significantly different from zero (i.e., baseline). One-sample *t*-tests comparing the change in MEP amplitude to zero revealed that the average change in amplitude was not significantly different from baseline at any of the post-iTBS time points (*p*'s > 0.117).

### 3.4. iTBS-Induced Plasticity in Bilateral Motor Cortices of LTP- and LTD-Like Responders

**Figure 4:**
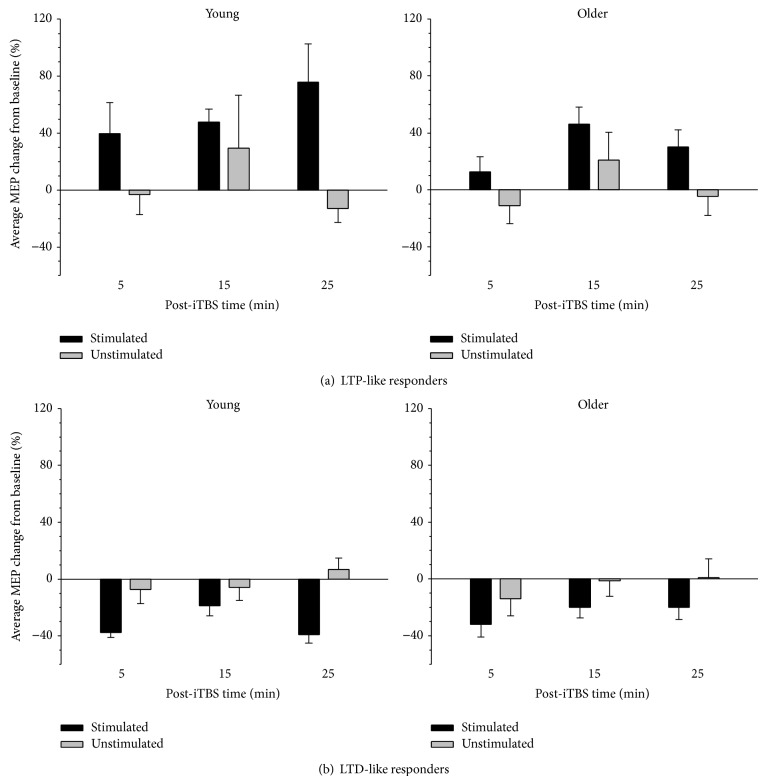
Average normalised MEP change following iTBS in young and older LTP-like (a) and LTD-like (b) responders. In both of the LTP-like and LTD-like responders, MEP change was comparable between young and older adults and was greater in the hemisphere receiving iTBS (stimulated) than the hemisphere not receiving iTBS (unstimulated). Error bars denote* SEM*.

As in numerous previous reports, the current study found substantial variability in individual responses to iTBS. Averaged across the time points after iTBS, 19 individuals showed an increase in MEPs (i.e., post-iTBS MEPs > 0%, young = 9) and were classified as LTP-like responders. Twenty-one individuals showed a decrease in MEPs (MEP < 0%, young = 11) and were classified as LTD-like responders. Data pertaining to these groups is depicted in Figure 4. As expected, the direction of MEP change varied as a function of response type, with LTP-like responders showing an increase in MEPs (Figure 4(a)) and LTD-like responders showing a decrease (Figure 4(b)). The difference between LTP- and LTD-like responders was confirmed by a significant main effect of response type; *F*(1,36) = 22.38, *p* < 0.001, and *ηp* ^2^ = 0.383. This effect, however, was much larger in the stimulated hemisphere of both groups (see Figure 4), as indicated by a significant interaction between hemisphere and response type: *F*(1,36) = 28.09, *p* < 0.001, and *ηp* ^2^ = 0.438. Follow-up paired comparisons demonstrated that MEP change differed significantly between the stimulated and unstimulated hemisphere in both the LTP-like (*t*(18) = 3.67, *p* = 0.002) and LTD-like responders (*t*(20) = 8.17, *p* < 0.001). Specifically, although MEP amplitude increased significantly relative to baseline in the stimulated hemisphere of LTP-like responders (*t*(18) = 4.88, *p* < 0.001) and decreased significantly relative to baseline in LTD-like responders (*t*(20) = 11.02, *p* < 0.001), MEP change in the unstimulated hemisphere did not differ significantly from baseline in either LTP-like (*t*(18) = 0.30, *p* = 0.766) or LTD-like (*t*(20) = 0.598, *p* = 0.557) groups. There was a weak trend toward an interaction between time, hemisphere, and response type; *F*(2,72) = 2.91, *p* = 0.070, and *ηp* ^2^ = 0.075, but no other main effects or interactions (all *p*'s > 0.183).

## 4. Discussion

The current study investigated whether plasticity manifests differently across the stimulated and unstimulated motor cortices in young and older adults following iTBS to the dominant (left) hemisphere. Contrary to our hypothesis, the effects of iTBS were found to manifest similarly in young and older adults, despite older adults exhibiting slightly attenuated cortical excitability at baseline (as indicated by an elevated TMS test intensity). Changes in MEP amplitude induced by iTBS, however, varied greatly across individuals, with only half the participants in each age group demonstrating the expected increase in cortical excitability.

### 4.1. Plasticity Induced by iTBS Is Subject to Large Individual Variation but Does Not Manifest in the Nonstimulated Hemisphere

Individual variability in iTBS effects has been demonstrated on numerous occasions [35–40]. In the current study, just under half the participants exhibited an increase in MEP amplitude in the target muscle (averaged across time) following iTBS. Consistent with one previous report [35], the proportion of LTP-like and LTD-like responders in the current study was similar in young (47% and 53%, resp.) and older adults (53% and 47%). There was also little difference in the proportion of males and females in the LTP-like (58% and 42%, resp.) and the LTD-like (43% and 57%) groups. These data suggest that neither age, gender, nor the allocation of spatial attention, which was controlled in the current study, explains the variability in our iTBS effects.

An important question in the current study was how plasticity manifests across the stimulated and unstimulated motor cortices. Previous studies suggest that iTBS induces an increase in cortical excitability in the stimulated hemisphere and a decrease in the unstimulated hemisphere [10, 26, 41]. In the current study, and as implemented in previous work, because cortical excitability at the group level did not differ reliably from baseline following iTBS, data were split into LTP-like and LTD-like responders [4, 35]. This analysis revealed significant plasticity in the stimulated hemisphere (an increase or decrease, resp.), but there was still no reliable change evident in the unstimulated hemisphere. One explanation for the inconsistency between this result and previous research is that iTBS was applied to the left M1 in the current study but to the right M1 in previous studies [10, 26, 41]. Some evidence indicates that the change in cortical excitability induced by brain stimulation and motor training varies depending on the dominance of the targeted hemisphere [42, 43], perhaps due to stronger inhibitory projections from the dominant to the nondominant motor cortex than* vice versa* [44].

Another explanation for differences between the current results and previous work relates to prior voluntary muscle activity. The intensity used for iTBS is typically set to a percentage of active motor threshold (aMT), which requires participants to maintain a voluntary contraction for a prolonged period before the iTBS intervention. Such activity can influence susceptibility to plasticity interventions and even alter the direction of effects ([45, 46], see [2] for review). The current study minimised any influence of prior voluntary muscle activity on iTBS-induced effects by basing stimulation intensity on resting excitability (rMT), as used successfully with iTBS in previous work [11, 46, 47]. Importantly, in the only study to investigate bilateral changes in cortical excitability after iTBS in young and older adults [26], the ipsilateral silent period was measured before and after iTBS. This measure requires participants to voluntarily contract the hand ipsilateral to the stimulation for a prolonged period, which may have altered responses to iTBS.

### 4.2. Manifestation of Plasticity across Bilateral Motor Cortices Is Not Altered in the Aged Brain

The results of the current study indicate that the magnitude and manifestation of plasticity induced by TMS are not altered in older adults. These findings are consistent with some previous studies [26, 35, 48] but are inconsistent with those reporting reduced plasticity in the motor cortices of the aged brain [3–5]. Though a possibility, there is no reason to expect that the sample of older adults tested in the current study differed systematically from those employed in previous studies of this kind. Therefore, the discrepancy between the results of the current study and those showing a reduction in TMS-induced plasticity in older adults may reflect methodological differences in the TMS protocol used. Specifically, most studies reporting reduced TMS-induced plasticity in older adults have implemented PAS [3, 4], which has been shown to target similar but not identical mechanisms to iTBS [49, 50]. Whereas PAS is based on spike-timing dependent plasticity and is dependent upon associative pairings of TMS and peripheral nerve stimulation [50], TBS is dependent on the rate of stimulation [27]. The observation of attenuated plasticity in older, relative to young, adults following PAS [3, 4], but maintained plasticity following iTBS [26, 35], suggests that different mechanisms targeted by these protocols may be more or less susceptible to age-related change.

In addition to methodological differences, the influence of attention has been largely overlooked in investigations comparing plasticity in young and older adults, despite evidence of attentional deficits with advancing age [29]. We have shown previously that LTP-like plasticity induced in the motor cortex by TMS is reduced under conditions of high attentional demand and when attention is directed away from the hand undergoing the plasticity-inducing procedure [30, 31]. Accordingly, the current study implemented a task that required participants to allocate their attention to the digit targeted by iTBS. An age-related reduction in plasticity was not found under these conditions. It is possible that by directing attention to the targeted digit iTBS-induced plasticity was restored in older adults. Future studies comparing the effect of iTBS when attention is directed to the targeted versus homologous digit of the opposite hand would clarify the degree to which plasticity in older adults is influenced by attention. If attention is more critical in maintaining plasticity in older compared to young adults, a greater change in plasticity would be evident when attention is directed to the opposite hand. If attention does not influence plasticity, however, there should be no difference between the two conditions.

### 4.3. Conclusions and Implications

The current study demonstrates that plasticity induced by iTBS is subject to large individual variability. The data indicate that although iTBS might be used to induce plasticity in the stimulated hemisphere, it is not associated with a manifestation of plasticity in the unstimulated hemisphere. Importantly, irrespective of whether participants demonstrated increased or decreased cortical excitability following iTBS, the manifestation of plasticity was not altered in older compared to younger adults. This finding suggests either that the mechanisms and/or pathways targeted by iTBS are not altered in the aged brain or that plasticity is enhanced in older adults when attention is directed to the digit targeted by the stimulation. Interestingly, the findings of the current study also suggest that strategies implementing iTBS to facilitate plasticity with the goal of enhancing motor function [51, 52] are likely to benefit young and older adults comparably. Critically, however, the factors determining an individual's response to iTBS, including the interaction between attention and age, require further investigation.

## Figures and Tables

**Table 1:** Mean and standard deviations for baseline cortical excitability.

	Young		Older	
	Stimulated	Unstimulated	Stimulated	Unstimulated
Baseline rMT (% machine output)	38.50 (*6.33*)	38.40 (*6.73*)	40.95 (*6.65*)	41.95 (*5.05*)
Baseline MEP amplitude (mV)	1.12 (*0.35*)	1.03 (*0.25*)	1.02 (*0.35*)	0.93 (*0.22*)
Test intensity (% machine output)	**46.80** (***7.95***)	**47.25** (***9.14***)	52.65 (*7.92*)	52.90 (*7.10*)

Note: test intensities (highlighted in bold) were significantly lower for young compared to older adults (*p* = 0.019). No other significant main effects or interactions were evident (all *p*'s > 0.125).
